# Stress in the social context: a behavioural and eco-evolutionary perspective

**DOI:** 10.1242/jeb.245829

**Published:** 2023-08-02

**Authors:** Kirsty J. MacLeod, Sinead English, Suvi K. Ruuskanen, Barbara Taborsky

**Affiliations:** ^1^ School of Natural Sciences, Bangor University, Bangor, LL57 2UR, UK; ^2^ School of Biological Sciences, University of Bristol, Bristol, BS8 1TQ, UK; ^3^ Department of Biological and Environmental Science, University of Jyväskylä, Survontie 9 C, FI-40014, Finland; ^4^ Department of Biology, University of Turku, Turku, FI-20014, Finland; ^5^ Division of Behavioural Biology, Institute of Ecology and Evolution, University of Bern, 3012 Bern, Switzerland; ^6^ Institute for Advanced Study, 14193 Berlin, Germany

**Keywords:** Hierarchy, Social behaviour, Social buffering, Stress, Transgenerational

## Abstract

The social environment is one of the primary sources of challenging stimuli that can induce a stress response in animals. It comprises both short-term and stable interactions among conspecifics (including unrelated individuals, mates, potential mates and kin). Social stress is of unique interest in the field of stress research because (1) the social domain is arguably the most complex and fluctuating component of an animal's environment; (2) stress is socially transmissible; and (3) stress can be buffered by social partners. Thus, social interactions can be both the cause and cure of stress. Here, we review the history of social stress research, and discuss social stressors and their effects on organisms across early life and adulthood. We also consider cross-generational effects. We discuss the physiological mechanisms underpinning social stressors and stress responses, as well as the potential adaptive value of responses to social stressors. Finally, we identify outstanding challenges in social stress research, and propose a framework for addressing these in future work.

## Teaser

**Summary:** We review the history of social stress research, and discuss effects of social stress within and between generations. We identify outstanding questions, and propose a framework for future work.

## Introduction

Since the inception of formal research on ‘stress’ in biology (Cannon, 1935; Selye, 1956), the social environment has been identified as one of the primary sources of challenging stimuli that can induce a stress response (see Glossary). ‘Stress’ can be defined as a process whereby an organism reacts to stressors, including detection of the stressor and the subsequent stress response (Wingfield et al., 1998; Taborsky et al., 2021). Stress is disruptive to homeostasis, the maintenance of the internal environment within life-sustaining limits via physiological mechanisms (the self-regulatory process of ‘allostasis’, achieved through allostastic mediators including hormones, cytokines and cardiovascular regulators; see Glossary) (McEwen, 2005). The stress response in vertebrates is characterised by activation of key physiological pathways: the sympathetic adrenal medullary system (SAM) and the hypothalamic–pituitary–adrenal (HPA) axis, which provide short- and long-term allostatic responses to stressors, respectively (see Box 1).

**Box 1. The HPA/I axis and SAM system**

When encountering stressors, individuals activate both the sympathetic adrenal medullary (SAM) system and the hypothalamic–pituitary–adrenal/interrenal (HPA/I) axis. Activation of the SAM system involves activation of the autonomous sympathetic nervous system, and, via sympathetic neurons, the excretion of adrenaline/noradrenaline from the adrenal medulla. Activation of the HPA/I axis causes the release of corticotropin-releasing factor (CRF) from the hypothalamus, which leads to the release of adrenocorticotropic hormone (ACTH) from the anterior pituitary, resulting in the release of glucocorticoids from the adrenal cortex or interrenal tissues. Importantly, the SAM system is a fast-acting system mediating stress responses within seconds, while the HPA/HPI vertebrate stress axis acts relatively slowly to stressors, in the range of minutes to hours. On detection of a stressor, sympathetic activation mediates a number of effects eliciting a ‘fight or flight’ response (increasing cardiovascular tone, respiratory rate, blood flow to skeletal muscles and blood glucose; Bauer et al., 2002). In addition, the connection between the sympathetic nervous system and the adrenal medulla leads to the release of stored catecholamines, primarily adrenaline and noradrenaline (Bauer et al., 2002). Typically, glucocorticoids exert negative feedback on both systems, restoring the organism to homeostasis. The SAM and HPA systems are anatomically and physiologically connected in the central nervous system (Sapolsky et al., 2000). It has been hypothesised that synchronised and symmetrical action of the two systems is required for optimal behavioural outcomes (Bauer et al., 2002).

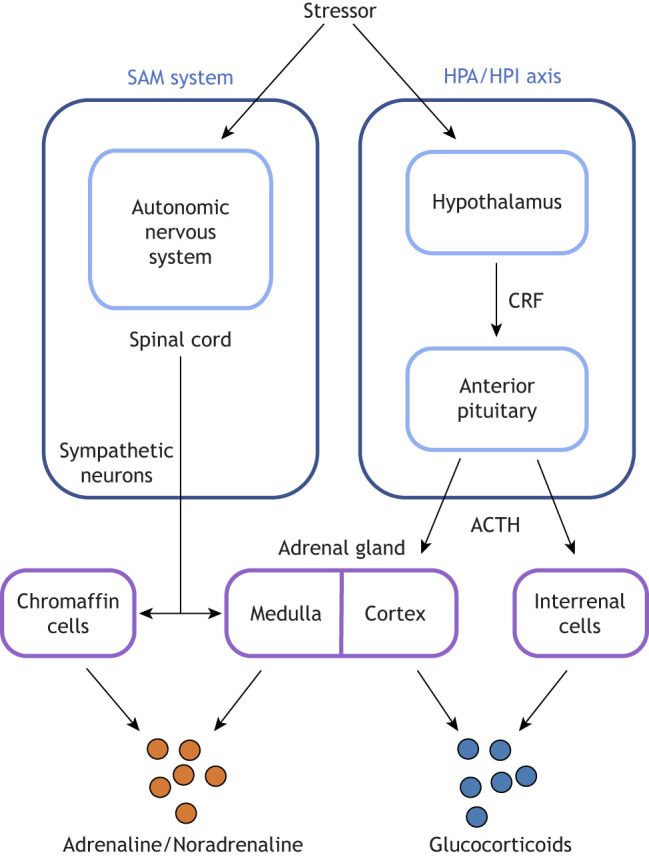

Potential stressors are wide ranging, and can include stimuli such as predation risk, low food availability and adverse weather events. In addition, an individual's social environment, which comprises interactions among conspecifics – including unrelated individuals, mates, potential mates and kin (Dantzer and Newman, 2022) – can act as a source of stressors (Table 1). The social environment is perhaps unique among stressors in that it also acts as a buffer from other sources of stress. Thus, stress in the social context encompasses stress derived directly from the social environment, as well as the role of the social environment in altering the outcomes of stress.

**Table 1.**
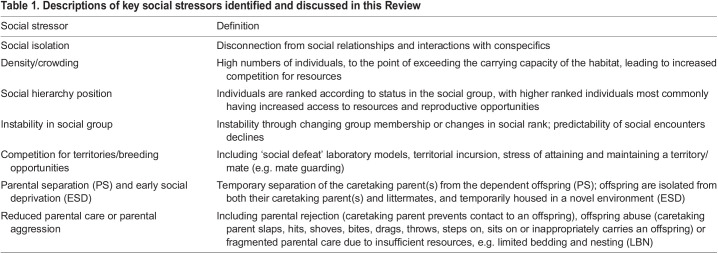
Descriptions of key social stressors identified and discussed in this Review

There are at least three reasons why stress in the social context is qualitatively different from non-social abiotic and biotic stress. First, the social domain is arguably the most complex and fluctuating component of an animal's environment*.* Social interactions differ not only in terms of the type of interaction (e.g. competitive, sexual) but also with respect to the identity of the interacting individuals, the number of social partners and their external and internal states (such as rank, size and emotional state). Social information has to be gathered quickly and may be incomplete, inherently generating uncertainty, which itself is an important stressor (Kaiser and Sachser, 2005). Moreover, social factors have varying time scales, from highly stable and predictable (e.g. long-term dominance hierarchies) to more rapid and unpredictable (e.g. aggression received from a conspecific depending on their current physiological condition).

Second, stress is socially transmissible*.* Social transmission of stress responses can be adaptive; for instance, transmission of a response to a predator can benefit an entire collective, as not all individuals may perceive the threat simultaneously, and a coordinated response can more effectively deter predators (e.g. Doran et al., 2022). However, if higher stress levels are transmitted across individuals within a social unit, short-term benefits, such as improved anti-predator responses, may be traded off against long-term costs of physiological stress such as oxidative damage (see Glossary; Noguera et al., 2017).

Third, stress can be buffered by social partners*.* Social factors have been mostly viewed as causes for social stress. However, the mere presence of a social companion (e.g. a mate, parent or another familiar member of the social group) can reduce the stress response in other individuals (DeVries et al., 2003; Culbert et al., 2019; Hennessy et al., 2009; Wu, 2021). There is also evidence that this ‘social buffering’ (see Glossary) can have a positive impact on measures of health and well-being (DeVries et al., 2003).

**Fig. 1.**
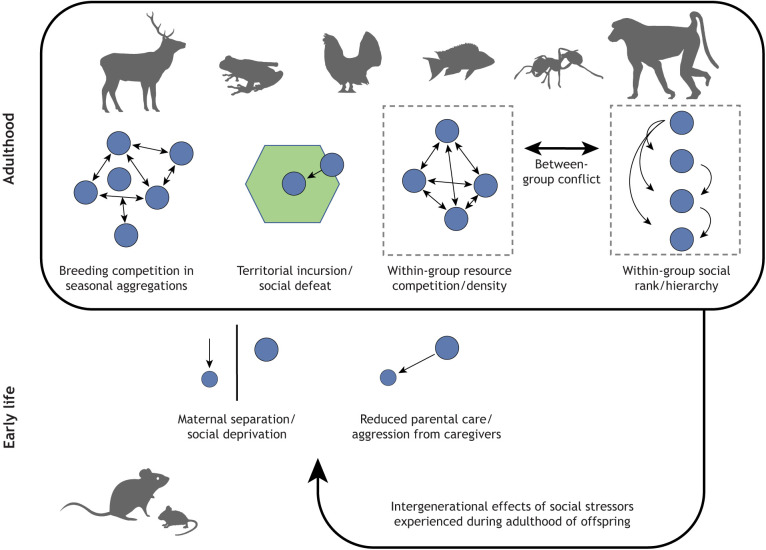
**Social stressors in animals at different life stages and across generations.** Some social stressors are experienced only in certain life stages (e.g. stress relating to maternal care is experienced only in early life, breeding competition is only experienced in adulthood). Stressors experienced during adulthood may influence the next generation through parental (intergenerational) effects. Blue circles represent individuals, and arrows depict the direction of stress. Silhouettes are from PhyloPic (https://www.phylopic.org/; Creative Commons).

Here, we review the history of social stress research, and discuss the effects of social stressors in three contexts: in early life, in adulthood and across generations (see Fig. 1). Individuals at different life stages can be exposed to different social stressors, but can also differ in their sensitivity to the same stressors. We highlight key experimental advances in the field that have furthered our understanding of physiological responses to social stress, and the potential for adaptive plasticity (see Glossary). We aim for a broad and diverse approach across taxa and social systems to illuminate the role of social stress in an eco-evolutionary context. We additionally highlight the outstanding challenges in social stress research and propose a framework for future work.

**Glossary**

**Adaptive plasticity**

Phenotypic changes in response to the environment (including the parental environment) provide fitness benefits.

**Allogrooming**

Cleaning or maintaining the body surface of a conspecific individual.

**Cooperative breeding**

Joint raising of offspring by their parents and non-parents (‘helpers’).

**Homeostasis/Allostasis**

Processes to help control the body's responses to an internal or external environmental stressor. Homeostasis: stability of physiological parameters for optimal functioning. Allostasis: process of maintaining stability of physiological parameters essential for life through changes the body makes in response to changing conditions.

**Match-mismatch hypothesis**

Early environmental influences (through parental effects or own experience) act as predictors for the later-life environment later so that, if the prediction is accurate, the offspring's phenotype ‘matches’ the environment in which it will live, increasing its fitness. If the prediction is wrong, there would be a ‘mismatch’ at the cost of the fitness of the offspring.

**Oxidative damage**

Damage (e.g. to cells and tissues) caused by an imbalance of free radicals and antioxidants in the body.

**Population cycling**

Populations undergo large, cyclic increases and declines in population size over a predictable period of time.

**Reaction norm**

A reaction norm depicts the range of phenotypes a single genotype can produce, depending on the environment.

**Social buffering**

The presence of a social partner moderates the stress response of an individual.

**Stress response**

The activation of coordinated neurophysiological responses in the brain and periphery to cope with environmental demands or stressors.

## A history of social stress research

Although stress research is now embedded into ecology and evolution (Boonstra, 2013a), it was long considered a subspecialty within medical sociology, primarily concerned with human health outcomes (Selye, 1956; Aneshensel, 1992). Consequently, early research on social stressors had its roots in human socio-psychology, particularly relating to disease or health parameters (Rahe et al., 1964). The concept of ‘social stressors’ in humans covers a broad list, including adverse social conditions (e.g. family instability: McEwen and Gianaros, 2010), unequal-power relationships (e.g. bullying, victimisation: Björkqvist, 2001) and social factors not directly related to physical interactions, such as wealth inequality (Wood et al., 2012; Wilkinson and Pickett, 2006). Just as Selye's (1956) pioneering early work in unpicking the basis of stress physiology noted a ‘general adaptation syndrome’ among patients presenting diverse conditions or challenges (Selye, 1950), this range of social stressors in humans has common outcomes, including obesity and metabolic disorders (Scott et al., 2012), impaired immune function (e.g. lower lymphocyte count: Owen et al., 2003; Dowd and Goldman, 2006), and depression and anxiety (Chaouloff, 2013; Tafet and Nemeroff, 2016).

Experimental work on social stress in humans has been largely correlational as a result of ethical constraints, but some replicable protocols have proven useful. For example, the Trier Social Stress Test (TSST) was designed to exploit the vulnerability of the stress response to socially evaluative situations, with participants asked to perform challenging tasks in front of a non-responsive audience (Kirschbaum et al., 1993; Allen et al., 2014). Application of the TSST has demonstrated biological responses to stress in human subjects, such as changes to glucocorticoid levels, immune function, cardiovascular function and sympathetic nervous system activity (reviewed in Allen et al., 2017).

There has been an impetus to understand effects of social stress on psychopathologies in humans (e.g. depression) through a better understanding of the underlying neurobiology and physiology (Chaouloff, 2013). However, the constraints of studying stress in humans experimentally have meant that laboratory studies using model organisms have been a key part of this research (Selye, 1956; Koolhaas et al., 1997). This has led to the development of experimental models of social stress in rats and mice (Koolhaas et al., 1997; Masis-Calvo et al., 2018), as well as several experimental paradigms to manipulate early-life stress (Van Bogedom et al., 2017; see Table 1). These experimental models have provided key insights into the role of the hypothalamic–pituitary–adrenal/interrenal (HPA/HPI) axis (Box 1; Wingfield et al., 1998, Bernier and Peter, 2001) in mediating social fear and agonistic interactions, as well as the physiological and behavioural consequences of chronic exposure to negative social experiences (reviewed in Masis-Calvo et al., 2018). Rodent models have been at the forefront of illuminating the potential for social buffering and its mechanistic underpinnings (Davitz and Mason, 1955; Beery and Kaufer, 2015). For example, pioneering experimental studies explored the effect of maternal licking and grooming on offspring stress reactivity, demonstrating long-lasting epigenetic effects occurring through increased glucocorticoid receptor expression in the hippocampus (Liu et al., 1997; Weaver et al., 2004). Primate models have also proved instructive, particularly in the context of social buffering through mother–infant bonding (Levine et al., 1997).

The opportunity to test these ideas in free-living animals was presented by an emerging body of behavioural ecology and socio-biology studies (Stuhrmann, 2022) that included significant work on social systems (e.g. Crook, 1970; Clutton-Brock, 1974; Clutton-Brock and Harvey, 1976; Gittleman, 1989) and the evolution of group living and cooperation, e.g. cooperative breeding (see Glossary) (e.g. Solomon and French, 1997; Stacey and Koenig, 1990; Hatchwell and Komdeur, 2000). This led to a boom in the study of social stressors in non-model organisms, including captive primates (Kappeler, 1993) and fish (Sørensen et al., 2013), as well as studies in the wild (e.g. baboons; Sapolsky and Mott, 1987). Although our understanding of the physiological consequences of social stressors in wild animals lags behind the work on laboratory rodents, the increased feasibility to accurately and relatively non-invasively measure, for example, endocrine profiles of wild animals in natural systems, has facilitated research linking social stress to animal physiology, in particular to HPA axis activity (Creel, 2001). Indeed, the comparative ease with which steroid hormones can be quantified relative to, for example, sympathetic catecholamines, coupled with the appealing spectrum of glucocorticoid effects on a range of physiological and behavioural parameters, have made them potentially an over-relied-upon metric of stress to date. Therefore, the bulk of vertebrate ‘stress’ research, including that on social stress, has focused on HPA axis activity alone (MacDougall-Shackleton et al., 2019; Sapolsky, 2021). However, recent years have seen an increase in more integrative studies including a broader range of measures of animal health (Snyder-Mackler et al., 2020; Lemonnier et al., 2022).

Studies of free-living systems have also considered social stress effects on key ecological phenomena, such as population cycling (see Glossary) through density effects (reviewed in Creel et al., 2013). More recently, ideas that maternal stress – including social stress – could adaptively programme offspring (as opposed to resulting in negative outcomes) in wild systems have gained traction (Sheriff et al., 2017). For example, an experimental increase in perceived density in red squirrel populations, and the associated stress response in breeding females, results in increased offspring growth, which is potentially adaptive if it allows young to outcompete conspecifics at high population densities (Dantzer et al., 2013).

## Social stress across the lifespan

### Early life

‘Early-life social stress’ refers to social stress experienced during the early postnatal period, when physiological systems are developing and are highly sensitive to perturbation. During early life, the brain undergoes a period of intense plasticity and maturation with long-term impacts on brain function and synaptic plasticity (Alves et al., 2022). The precise beginning and end of ‘sensitive periods’ differs among organisms and depends on juveniles’ environmental experience (Michel and Tyler, 2005; Panchanathan and Frankenhuis, 2016). Here, we mostly focus on social stress occurring shortly after birth, but generally consider the period until the onset of sexual maturation. Although early-life social stress most often has immediate effects on behaviour and physiology, effects can also persist into the later juvenile stage or adulthood (Snyder-Mackler et al., 2020). We focus primarily on these long-term effects, given that they are likely to be relevant to eco-evolutionary dynamics.

Relevant early-life social stressors can be categorised as relating to variation in parental care or to variation in the number of social interactions with family or group members, both of which are discussed in more detail below (see Fig. 1). We also discuss potential programming of social behaviour in response to early-life social stress.

#### Variation in parental care

Multiple experimental paradigms have been developed for mammalian laboratory models to induce aversive early social environments and study their effects on phenotypes (e.g. Sandi and Haller, 2015, Van Bogedom et al., 2017). Studies have focused on manipulating the quality and quantity of maternal care to mimic naturally relevant variation in care and to induce stress in dependent offspring. Even within a single species, there is variation in the amount or quality of parental care: in Norway rats (*Rattus norvegicus*), for example, there is considerable variation in the quality of maternal care provided, measured as licking and grooming and arched-back (as opposed to normal posture) nursing (Meaney, 2001). Offspring of mothers providing lower quality maternal care (reduced licking/grooming and normal posture nursing) express a lifelong higher stress reactivity compared with those from mothers who provide higher quality care (Liu et al., 1997). As adults, they become mothers which themselves provide lower quality care, thereby transmitting the effect of their own early-life experience to the next generation (Francis et al., 1999; Champagne and Meaney, 2007).

In rodent and primate models, a range of studies have shown that the ‘quality’ of maternal care affects a suite of later behaviours in offspring. Experimentally separating rodent offspring from their mothers (‘separation’) or mothers and broodmates (‘deprivation’) for short periods simulates interrupted maternal care or maternal neglect (Zimmerberg and Sageser, 2011; Van Bogedom et al., 2017). Short maternal separations of up to a few hours typically increase fearful behaviour and impair memory performance (Tractenberg et al., 2016; Sachser et al., 2018), whereas longer periods of separation can enhance fear memory and synaptic plasticity in adult offspring (Oomen et al., 2010). Similarly, deprivation can induce a submissive phenotype in adults, which is inferior to more dominant phenotypes in competition over critical resources (Benner et al., 2015). In rhesus macaques (*Macaca mulatta*), effects of maternal rejection – whereby mothers deny contact to offspring – were investigated by cross-fostering infants of mothers that had historically rejected their infants with mothers that had not displayed rejection (Maestripieri et al., 2007). In adulthood, females exhibited rejection rates similar to those of their foster mothers; however, contact-seeking behaviour with their daughters more closely resembled that of their biological mother (Maestripieri et al., 2007), suggesting that both early-life social stress and genetic background contribute to behavioural phenotypes as mothers.

Social stressors involving reduced maternal care typically result in stronger responses of the HPA axis towards stressors experienced during later life (Sandi and Haller, 2015; Van Bogedom et al., 2017; Banerjee et al., 2012). This hyper-reactivity is caused by increased production of stress-induced corticosteroids, typically mediated by increased corticotropin-releasing hormone signalling and reduced negative feedback through glucocorticoid receptors (Van Bogedom et al., 2017). However, there is also contradictory evidence showing that the HPA axis is downregulated after early social deprivation in both rodents (Sandi and Haller, 2015; Van Bogedom et al., 2017) and birds (Goerlich et al., 2012). The baseline activity of the vertebrate stress axis can also be affected permanently by early-life social stress, as shown in the cichlid *Neolamprologus pulcher*, which has lower baseline cortisol levels in adulthood after being socially deprived of parents and helpers in early life (Antunes et al., 2021).

An extreme early-life social stressor that also exhibits natural variation is aggression towards dependent offspring from caregivers or other group members (Kinnally et al., 2010). This is particularly striking in the form of naturally occurring abusive behaviour by mothers (Table 1). Occurrences of abusive behaviour are often preceded by stressful social events such as intra-group aggression (Maestripieri, 1994). Abuse by rodent mothers can be experimentally induced by the repeated presence of a male intruder (Nephew and Bridges, 2011). Such abuse then induces fearful behaviour in offspring, which develop abnormal maternal behaviour later in life (Carini and Nephew, 2013; Murgatroyd and Nephew, 2013). In rhesus macaque offspring, experiencing abusive maternal behaviour has been associated with subsequent increased responses to threat (Mandalaywala et al., 2014), increased fearful behaviour (Howell et al., 2014, 2017) and increased contact-seeking behaviour (McCormack et al., 2006). A cross-fostering experiment showed that offspring abused by adoptive mothers in early life are more likely to abuse their own infants, regardless of the abusive behaviour of the biological mother (Maestripieri, 2005). Although the consequences of abusive parental behaviour in mammalian offspring are mostly considered to be maladaptive, potential adaptive consequences of parental aggression have also been documented. For example, male stickleback, which guard and tend eggs and small offspring, chase away their offspring a few days after hatching in habitats with predators (but not in the absence of predators), which is thought to prime anti-predator responses in the young (Huntingford et al., 1994). Similarly, pup ‘shoving’ by naked mole-rat parents increases in experimentally disturbed environments, and shoved pups are subsequently more likely to flee from danger, a possibly adaptive consequence (Stankowich and Sherman, 2002).

#### Social interactions with other group members

Parents do not form the only source of social stress in early life, and other aspects of the social environment can shape the stress response and affect later phenotype. Social stress can be elicited by increasing group density, for example, which enhances resource competition. Brood size enlargements are a common experimental paradigm for considering downstream effects of the early social competitive environment. For instance, zebra finches reared in experimentally enlarged broods have reduced growth and cell-mediated immunocompetence (Naguib et al., 2004), and brood size is also commonly associated with nestling glucocorticoid levels across avian species (e.g. Gil et al., 2008; Vitousek et al., 2017). Increased rearing density also affects later behaviour in guppies, leading to poorer social learning skills when locating food and reduced shoaling tendency (Chapman et al., 2008), both of which are assumed to negatively affect fitness.

Social abilities can also be affected by reduced opportunities for social contact during early development. Mice can be reared in communal nests (in which there are multiple mothers and broods) or in single-mother nests. In single-mother nests, pups receive less maternal care and fewer peer-to-peer interactions than in communal nests; in adulthood, these mice are less resilient to social stress induced by social instability (see Table 1; Branchi et al., 2013). Opportunities for social interactions can also be decreased by reducing the number of carers. For instance, in zebra finches, birds raised only by a father instead of a pair show a stronger physiological stress response to isolation in adulthood (Banerjee et al., 2012). Likewise, deprivation of peers during the juvenile period reduces the ability of rhesus monkeys to express adequate social behaviours: deprived monkeys show more submissive and socially anxious behaviour, and socially incompetent, aggressive behaviour in non-threatening social situations (Kempes et al., 2008, 2009). Offspring of cooperatively breeding cichlid fish, *N. pulcher*, reared only among their siblings generally have fewer social interactions among peers during rearing, and in the late juvenile phase and during adulthood they behave less appropriately in competitive contexts compared with offspring reared with parents and helpers (Arnold and Taborsky, 2010; Nyman et al., 2017; Taborsky et al., 2012).

#### Early life social stress and adaptive plasticity

The long-term effects of early-life social stress on behaviour and physiology strongly suggest that the phenotype of an individual can be ‘programmed’ during the earliest stages of development (e.g. Spencer, 2017; Taborsky et al., 2021). For instance, HPA/HPI axis programming can be induced by direct exposure to either cortisol or a glucocorticoid receptor blocker during early life; in the cichlid *N. pulcher*, this has been shown to result in differential abilities to express adequate social behaviour as juveniles (Reyes-Contreras et al., 2019) and to solve a learning task in adulthood (Reyes-Contreras and Taborsky, 2022). From an evolutionary perspective, programming of the social behavioural phenotype in response to early-life social stress (resulting in, for example, increased social fearfulness and reduced social competence, social learning skills and sociability or even the development of abusive behaviour towards their own offspring) may negatively impact fitness. The ‘cumulative stress hypothesis’ posits that individuals are more likely to suffer from fitness reduction as adversity accumulates over life (Nederhof and Schmidt, 2012). However, several related hypotheses propose that developmental plasticity in response to stress can be adaptive; for example, by preparing individuals for future stressful challenges in their environment in a way that maximises fitness (Hales and Barker, 1992; Gluckman et al., 2005; Love and Williams, 2008). This kind of adaptive behavioural adjustment should also be expected to occur in the social domain where animals undergoing early-life stress show enhanced fitness when coping with later-life stressors; however, examples relating to the social domain remain scarce.

### Adulthood

Adulthood, often defined in animals by the onset of sexual maturation, is a critical stage for realising reproductive potential and is therefore important in determining ultimate individual fitness. Here, we focus on several well-studied social stressors that are particularly or exclusively pertinent in adulthood, such as competition with conspecifics for breeding resources and interactions related to the social hierarchy (see Fig. 1).

#### Competition

##### Social defeat and territoriality

Much of the early work on social stress during adulthood has been led by research in laboratory rodent models following the ‘social defeat’ protocol (Miczek, 1979). This protocol mimics territorial incursion in natural settings, being based on the establishment of a territory by a resident individual and the introduction of an intruder (Miczek et al. 1990). Social defeat induces significant physiological effects in rodent models, including changes to blood pressure and body temperature, and activation of the HPA axis, as shown by increases in plasma levels of adrenocorticotropic hormone (ACTH) and corticosterone (Bohus et al., 1987; Koolhaas et al., 1997; Miczek et al., 1990). Research on social defeat in laboratory systems has also demonstrated how prior experience of social stress can determine future social behaviour (Kudryavtseva, 1991, 2000), which is likely to be through the effects of chronic stress on dysregulation/hyperactivation of the HPA axis (Sapolsky, 2002) and breakdown of inhibitory feedback mechanisms (Jacobson and Sapolsky, 1991; Bartolomucci et al., 2003, 2004).

Consequences of territory incursion have been studied in free-living animal systems, from the non-social to the highly cooperative. Simulated territorial incursion experiments in wild birds – for example, using playback of unfamiliar song – have demonstrated consistent positive effects of perceived intrusion on baseline glucocorticoid levels (Silverin, 1993; Landys et al., 2010; Pinxten et al., 2004). The social stress of territorial incursion may scale up to group level in species with more complex social environments; for example, simulated incursion in cooperatively breeding white-browed sparrow weavers (*Plocepasser mahali*) results in increased aggressive behaviours from all group members (Wingfield and Lewis, 1993). Additionally, the stress of between-group conflict can lead to reduced fitness: groups of *N. pulcher* cichlids that are experimentally ‘intruded’ upon appear to reduce their investment in eggs and suffer reduced offspring survival (Goncalves and Radford, 2022). Furthermore, between-group conflict can feed back to intragroup dynamics; for example, allogrooming (see Glossary) can occur at a higher rate following conflicts of greater intensity (Schino et al., 1998; Radford, 2008).

##### Competition during breeding

Competition during breeding is another prominent social stressor during adulthood. Breeding is linked to peaks in population density in many non-group-living species, which is combined with an increase in mate competition and direct agonistic encounters. Early work in captive small mammal systems found associations between peaks in density with aggressive interactions and an increase in adrenocortical activity (Christian, 1950, 1971). Similar relationships between population density and adrenocortical activity have since been demonstrated in wild mouse and vole populations (Boonstra and Boag, 1992; Novikov and Moshkin, 1998; Harper and Austad, 2004); however, no effects of density (*Microtus pennsylvanicus*; Edwards et al., 2023) or opposite effects (density negatively correlates with glucocorticoids) have also been seen (*Microtus ochrogaster*; Blondel et al., 2016).

Cues that signal the presence of rivals can also act as social stressors, generating physiological responses without the need for physical interaction. In green treefrogs (*Hyla cinerea*), as in many anurans, acoustic signalling is a key part of sexual behaviour; in this species, corticosterone levels are elevated by hearing a chorus (Burmeister and Wilczynski, 2000), and they are higher in males that lose vocal contests in natural choruses compared with contest winners (Leary, 2014), with consequences for subsequent reproductive success (Leary and Crocker-Buta, 2018). In addition, agonistic signals spanning multiple sensory modalities stimulate the production of stress hormones in rival males in several other species (Dunlap et al., 2013; Schubert et al., 2009; Yang and Wilczynski, 2003).

Once attained, retaining and restricting access to mates can be a further stressor. Mate guarding is associated with increased glucocorticoid levels in several primate species (Bergman et al., 2005; Girard-Buttoz et al., 2014). In invertebrates, mate guarding is thought to incur costs – for example, Japanese beetles (*Popillia japonica*) appear to suffer energetic and thermoregulatory costs (Saeki et al., 2005); however, in invertebrates, there has been less focus on hormonal markers of stress. Finally, being constrained to pair with a poor-quality mate may also be a source of social stress: female Gouldian finches (*Erythrura gouldiae*) experimentally paired with poor-quality mates have 3–4 times higher circulating corticosterone levels than those observed in females that are paired with preferred mates (Griffith et al., 2011).

#### Social hierarchy

Social hierarchies are of particular importance during adulthood because of their consequences for breeding and survival. Primate researchers began reporting correlations between biomarkers of stress, such as glucocorticoid hormones and social rank, in non-human primates in the 1980s (Eberhart et al., 1983; Sapolsky, 1989), and investigation of hierarchy effects in a broad range of group-living species has followed, including continuing work across (but not limited to) primates (reviewed in Abbott et al., 2003; Simons and Tung, 2019), humans (reviewed in Sherman and Mehta, 2020) and fish (e.g. Ejike and Schreck, 1980; Dara et al., 2022; Bessa et al., 2021).

Early work matched intuitive expectations that low-ranking individuals suffer greater effects of social stress related to hierarchy position (such as hyperactivity of glucocorticoid system, enlarged adrenals and impaired sensitivity to negative-feedback regulation: Eberhart et al., 1983, Sapolsky, 1989, 2005). In addition to glucocorticoids, there has been experimental exploration of effects of social hierarchy-related stress on a variety of health parameters (e.g. heart disease in monkeys: Petticrew and Davey Smith, 2012; wound healing in baboons; Archie et al., 2012). Neurobiological changes resulting from hierarchy position have also been observed, such as apical dendritic atrophy of hippocampal CA3 pyramidal neurons in subordinate tree shrews (Magariños et al., 1996) and increased neurogenesis in dominant rats in certain brain regions (Kozorovitskiy and Gould, 2004). Differences between individuals in health outcomes as a result of social rank may be linked to tissue-specific changes in gene regulation, such as in genes related to inflammation (Tung et al., 2012; Simons and Tung, 2019). For example, in rhesus macaques, social rank predicts immune cell proportions, and gene expression response to immune challenge such that low-ranking females display a stronger inflammatory response to bacterial challenge (Snyder-Mackler et al., 2016).

We now understand that social stress effects related to hierarchy position are considerably context dependent, being affected by factors such as dominance style, hierarchy stability and availability of social support (Sapolsky, 2005). In general, when hierarchies are relatively stable, maintained through non-physical intimidation, and subordinate individuals are exposed to relatively high-frequency stressors and cannot evade dominant individuals, subordinate individuals show higher levels of physiological stress (Eberhart et al., 1983; Abbott et al., 2003). Across 22 fish species, for instance, subordinate individuals had higher basal cortisol than dominant fish, and this effect was stronger in small versus large groups (Bessa et al., 2021). However, when dominant individuals must repeatedly and aggressively assert their rank, as is the case, for example, in cooperative breeders and in species with transient periods of rank instability, dominant individuals show higher levels of physiological stress (Abbott et al., 2003; Sapolsky, 2005; Creel, 2022). A study of captive rhesus macaques showed that high-ranked monkeys with more ambiguous status had higher levels of inflammation than low-ranked monkeys (Vandeleest et al., 2016). In humans, unstable high status is associated with greater cortisol reactivity and slower recovery to baseline levels than observed in those with stable high status (Knight and Mehta, 2017).

Sex differences in the link between social hierarchy and stress have also been found. A systematic review of stress, social hierarchies and heart disease in monkeys found that in females, a more dominant status seems to buffer individuals from the negative consequences of stress, whereas the reverse is true in males (Petticrew and Davey Smith, 2012). Likewise, in some cooperative carnivores, hierarchy effects on basal and stress-induced glucocorticoid levels are dependent on sex. Basal urinary/faecal glucocorticoids are positively correlated with rank position in female dwarf mongooses and African wild dogs, while in males there is no correlation, or a weaker relationship (Creel, 2022). At the same time, stress-induced glucocorticoid levels are higher in dominant female dwarf mongooses, while the reverse is true in males (subordinate levels exceed those of dominants; Creel, 2022).

Finally, social buffering is also important in mediating the effects of social stress associated with hierarchical position. A meta-analysis of hierarchy effects across primates demonstrated that opportunities for social support significantly influence the relationship between social rank and stress physiology (Abbott et al., 2003). Specifically, subordinates experiencing more stressors have higher relative levels of cortisol, yet this effect is diminished when they have social support (i.e. when social contact is available) (Abbott et al., 2003).

#### Adulthood social stress and adaptive plasticity

Adulthood is generally not considered as sensitive a period for plasticity as early life (or juvenile development); nevertheless, there is still scope for adaptive plasticity in response to social stressors in adulthood. For example, physiological changes induced by social stress contribute to hierarchy maintenance or establishment, thus determining the long-term social circumstances of an individual. This is illustrated in rodents, in which the stress of social defeat results in substantial, permanent changes in brain neurochemistry (Blanchard et al., 2001). Laboratory studies of rats have shown that in dyadic contests, stress experienced by one of the individuals before their first encounter influences both the rank achieved during the encounter and the capacity to retain long-term memory for the achieved hierarchy (Cordero and Sandi, 2007; Timmer and Sandi, 2010). This supports the idea that social stressors can induce adaptive changes to behaviour through neuronal plasticity. Testing this in more ecologically relevant systems and non-model organisms is challenging, but potentially a rich area for further study (Lemonnier et al., 2022).

### Intergenerational effects of social stress

‘Intergenerational social stress’ occurs when the social environment and experiences of the parental or grandparental generation influence the phenotype of the offspring (Perez and Lehner, 2019). The earliest studies of intergenerational effects of social stress date back to the 1960s, when Keeley (1962) reported that the density (or crowding) of mothers influenced offspring behaviour in rodents. Social stress in a cross-generational context has been subsequently studied in relation to parental crowding/density, social isolation, social confrontation or changing social hierarchy/social group. A key feature of most studied stressors to date is social instability, which leads to low predictability of the social environment (Kaiser and Sachser, 2005). Because of the limited number of studies, here, we have generalised over different types of social stressors in our discussion of intergenerational social stress.

#### Maternal social stress

Most of the intergenerational social stress research to date has focused on maternal social stress, and has been largely dominated by studies in humans and captive model species (Kaiser and Sachser, 2009). The general conclusion from these studies is that offspring from prenatally socially stressed mothers display markedly greater ACTH and glucocorticoid responses to physical (e.g. immune challenge) and psychological (e.g. restraint and elevated platform exposure) stressors in adulthood compared with control offspring (Abe et al., 2007; Bosch et al., 2007; Brunton and Russell, 2011). Yet, for basal glucocorticoid levels, the data are equivocal: maternal social stress has been reported to cause both decreased and increased basal glucocorticoid levels in offspring, and also to have no effects (Babb et al., 2014). Data from social stress in production animals show similar patterns. For example, in pigs, offspring from socially stressed mothers display increased and more prolonged cortisol responses compared with those of controls (Jarvis et al., 2006), and higher maternal stocking densities in goats increases offspring fearfulness (Chojnacki et al., 2014). Prenatal social stress in mothers can be mediated by direct transfer of glucocorticoids (or other hormones) through the eggs or placenta, as is known to be the case for other stressors (Groothuis et al., 2019). However, there may be taxonomical differences, caused by, for example, differences in placenta structure between species (e.g. Chojnacki et al., 2014). A putative mechanism by which maternal (and paternal) social stress may affect offspring phenotypes is through epigenetic changes (in sperm or ova), such as changes in DNA methylation, small RNAs or histone modifications. Maternal social stress has been found to increase glucocorticoid receptor gene methylation in offspring, resulting in reduced glucocorticoid receptor density (i.e. higher levels of circulating glucocorticoids) (e.g. psychosocial stress associated with war-related events: Mulligan et al., 2012; meta-analysis: Palma-Gudiel et al., 2015; review in humans: Martin et al., 2022).

There are fewer data on intergenerational social stressors in non-mammals; in birds, the effects of maternal social stress on offspring stress physiology and behaviour are inconclusive. For example, Langen et al. (2019) showed that in Japanese quail (*Coturnix japonica*), maternal group size has no effect on offspring glucocorticoid response, whereas in gulls (*Larus fuscus*), parental density affects offspring social behaviour (Salas et al., 2022). Furthermore, zebra finch offspring from parents where mothers experienced pair separation and re-pairing are less behaviourally responsive to isolation (vocalisations and perch hops: Schweitzer et al., 2014), and, in quail, maternal social instability increases offspring emotional reactivity (e.g. increased latency to emerge from a shelter, stronger reaction to separation: Guibert et al., 2010). The effects of transgenerational social stress may also be sex dependent: in chickens, male, but not female, offspring of females experiencing social isolation in early life show a dampened glucocorticoid response (Goerlich et al., 2012). In piscine models, it is well known that parental crowding influences offspring phenotype, such as size (e.g. McCormick, 1998, 2006). Yet, the effects on fish physiological stress responses are relatively less studied so far: in a rare study, dominance hierarchies in zebrafish (*Danio rerio*) mothers led to the larvae of dominant mothers exhibiting significantly lower baseline cortisol levels and expression of HPI-related genes than offspring of subordinate females (Jeffrey and Gilmour, 2016). To our knowledge, data on the intergenerational effects of social stressors on offspring behaviour or stress responses in reptiles or amphibians are not available, although other maternal stressors are known to influence offspring stress physiology and behaviour through increased corticosterone deposited in eggs (e.g. Warner et al., 2009; Ensminger et al., 2018; Polich et al., 2018). In invertebrates, density appears to influence intergenerational social stress; for example, in locusts, eggs from solitary animals show higher expression of (cold) stress-related genes compared with those from gregarious animals (Wang et al., 2012), yet data on social effects on stress responses in invertebrates are very scarce.

#### Paternal social stress

While consequences of maternal social stress are extensively studied, it is only in the last decade that the potential role of paternal social stress on offspring stress axis and behaviour has been investigated. The first observations of effects of the paternal social environment came from studies of children of survivors of the Second World War (Rosenheck, 1986). However, these studies cannot often separate postnatal from prenatal effects. A recent review (Cunningham et al., 2021; see Table 1) summarises current knowledge on the effects of paternal social stress on offspring in mammals (humans and rodent models), and concludes that social stress in the form of paternal early-life social isolation and paternal social defeat can influence offspring stress physiology; the effects are mostly seen on male offspring and tend to depend on the father's exposure duration (e.g. Franklin et al., 2010; Dietz et al., 2011; Gapp et al., 2014; Gapp and Bohacek, 2018). Paternal density was found to influence offspring fitness in semi-wild rodents, but the underlying mechanisms are not fully understood (van Cann et al., 2019).

In sum, both maternal and paternal social environment can have long-lasting carry-over effects on offspring behaviour and physiology, yet the relative strength of maternal or paternal social stress on offspring has not been investigated. Paternal chronic stress, in terms of both social and non-social stressors, influences paternal sperm microRNAs, and reduces HPA axis responsivity in offspring (Rodgers et al., 2013). Kong et al. (2021) recently reported how paternal social instability, which is known to cause sex-specific effects on anxiety in mice, leads to systematic changes in DNA methylation of four key genes (*Bdnf*, *Adora*, *GATA* and *Itpr3*) in paternal sperm, in the blastocyst and in offspring hypothalamus. These genes are known to be linked to stress and anxiety.

#### Intergenerational social stress and adaptive plasticity

A key question arising from the studies discussed above is whether intergenerational plasticity in stress responses can increase offspring fitness (e.g. predictive-adaptive response hypothesis: Gluckman et al., 2005). Despite much interest, conclusive evidence for adaptive effects remains scant. A series of elegant studies in guinea pigs and in wild cavies (reviewed in Kaiser and Sachser, 2009; Siegeler et al., 2011, 2017; Sangenstedt et al., 2018) demonstrated that social instability during pregnancy causes behavioural and neuroendocrine masculinisation in daughters and a less pronounced expression of male-typical traits in sons. It was further hypothesised that such behavioural effects of maternal social stress may be adaptive: social instability is likely to occur in high-density populations, in which highly competitive (masculinised) females would cope better, whereas in the same high-density populations, less-competitive males that could delay reproduction might achieve higher success in the long run (match-mismatch hypothesis; see Glossary). The hypothesis was tested for both female and male offspring (Siegeler et al., 2017; Sangenstedt et al., 2018), and the results suggest that offspring have lower cortisol levels and higher weight gain in social environments matching their maternal social environment. However, the design of this experiment was not fully factorial, as offspring from unstable and stable maternal environments were only reared in stable postnatal environments. Therefore, we cannot exclude the potential for a ‘silver spoon’ effect, which occurs when offspring of mothers in good condition (those coming from a stable maternal environment) have fitness benefits over those of offspring of mothers in poor condition. Furthermore, the generality of such effects in different taxa needs to be further studied.

## A framework for future research

Our Review highlights several challenges that have thus far limited our knowledge and interpretations of social stressors and their effects throughout the lifetime. Here, we describe these challenges in more detail and, where possible, suggest a course for future work to address them.

Despite the rich history of studying the effects of environmental stressors across a wide variety of species, defining ‘stress’ itself has been called ‘a futile exercise’ (Levine, 2005) because of inconsistencies in terminology (e.g. McEwen, 2005; Koolhaas et al., 2011), clashing frameworks (e.g. Day, 2005; Kültz, 2020) and an over-reliance on taxon-specific physiological markers that are not synonymous with ‘stress’, i.e. the glucocorticoid hormones (MacDougall-Shackleton et al., 2019). Given the prevalence of social stressors across taxa, these challenges arise when trying to find a common framework applicable across groups, particularly when linking vertebrates with invertebrates. Definitions and frameworks that enable us to draw comparisons across a broader range of taxonomic groups are necessary (though difficult to achieve).

Data from a broad range of social and non-social species (i.e. species with different life histories) are needed to understand the full complexity of the effects of social stressors. Such data could also provide the basis for formal comparative analyses on the evolution of the stress response system across taxa, and link this to levels of social organisation. Although data from humans and captive model species are abundant, and can inform us on the mechanistic underpinnings of social stress, species that exhibit different levels of sociality should be explored (e.g. group living, biparental polygamous or biparental, solitary). Laboratory rodent data are also generally biased towards males, but there is evidence for sex-specific effects (e.g. Beery and Kaufer, 2015). However, we acknowledge that a taxonomically diverse approach comes with the difficulty of identifying species-specific stressors and determining responses to social stressors; the physiological responses across taxa, especially when comparing vertebrates and invertebrates, differ hugely (see above).

Even within the field of vertebrate stressors, more work in natural systems is needed, and extrapolating results from model organisms to the natural world should be done cautiously. Importantly, laboratory models often refer to chronic stress protocols, whereas in the natural world, the existence of similar chronic stress has been debated (Creel et al., 2013; Boonstra, 2013b). Furthermore, under laboratory conditions, variation in the strength or severity of stressors has been often ignored: available data from model organisms refer almost exclusively to the effect of strong social stressors, and the severity of social stress is rarely considered as a variable (Zelena et al., 1999). Recent studies have included ‘mild’ stress protocols to increase the variability, yet in natural populations a wide range of social stressors exists (e.g. the level of competition, harassment or parental care quality). Therefore, in future studies, experimental designs that vary the severity, temporal pattern (e.g. acute versus chronic) and type of social stress should be considered. Finally, the behavioural paradigms used in laboratory models (e.g. the social defeat model of territorial incursion) are potentially restrictive and limited in their ecological validity. We need to develop more ecologically relevant protocols and perform critical evaluation of the currently available evidence.

There are multiple areas where ecological studies could be developed based on evidence from laboratory studies. In wild populations, studies have largely focused on describing associations between different social stressors and the HPA axis. However, to understand the fitness consequences, eco-evolutionary research needs to take a broader view of different physiological pathways associated with social stressors (Lemonnier et al., 2022).

There are three ways in which a broader perspective on the links between social stress and animal physiology could be achieved. First, HPA axis research should be further expanded from simple measures of circulating hormones to also consider binding globulins and receptors, and their possible epigenetic modifications, which could generate long-term effects of stressors (e.g. Liu et al., 1997; Weaver et al., 2004). Second, the function of the HPA/HPI stress response and that of the fast-acting SAM system seem to be intricately connected; thus, the two stress response systems should be considered concurrently (Box 1; Bauer et al., 2002). The SAM system has rarely been considered in the context of social stress, especially in studies of natural populations. Furthermore, the HPA/I axis and SAM systems also interact with other endocrine and neurohormone systems, such as nonapeptide pathways (Smith and Vale, 2022), and therefore understanding the systems-level interactions is crucial. The ongoing development of omics approaches is a potential way to address such questions. Third, we need to study a wider range of physiological, neurological and behavioural mechanisms in wild populations. Such mechanisms have been thoroughly studied in model organisms, where it has been shown how cellular metabolism, oxidative stress, cellular senescence, immunity, brain function and the regulation of biological rhythms are key physiological responses affected by social stress, sometimes independently of the HPA axis response (Lemonnier et al., 2022). Broadening our approach in this way would help us to better understand the pathways mediating the effects of social stress and buffering on individual health and population demography. Although several of these measurements are challenging in wild populations for practical or ethical reasons, technical advancements in bio-logger technologies for behavioural responses now provide new frontiers for behavioural and physiological research in the field of social stress.

Additionally, both laboratory and wild population studies tend to focus on measuring mean values of responses to social (or non-social) stressors; individual variation and reaction norm (see Glossary) approaches (e.g. Hau et al., 2016) have rarely been applied. In other fields of stress physiology, the flexibility of the endocrine axes is increasingly being addressed (Lemonnier et al., 2022), and it provides important knowledge regarding adaptations. Such an approach would be a fruitful avenue for social stress research.

The predictability or reliability of the social environment as a cue may differ across taxa; this will influence the adaptive value of social cues and the evolution of responses to social stressors. Moreover, data on these important factors in natural environments are scarce (Taborsky et al., 2021). Although the lack of data on predictability also exists for many non-social environmental factors, obtaining such data in natural systems in the social domain is inherently challenging: an individual's responses to social stressors depend on the behaviour of other individuals in a society, which will be to some degree unpredictable, and which can either enhance or buffer stress responses.

The outcomes of social stressors or social buffering, even within species, depend on environmental context, social organisation and the characteristics of the group or individual (e.g. novel or familiar conspecifics; Beery and Kaufer, 2015). Thus, thorough and complex experimental designs are needed to tease out their effects. Furthermore, the field would benefit from better integration with ecology to aid us in understanding the outcomes of social stress on populations and their demography or growth trajectories (for example, through theoretical modelling of social stress). Previous studies have considered animal intergroup conflicts, dating back to the classical hawk–dove models (Maynard Smith and Price, 1973), but the models mainly refer to social conflicts, and the effects of social buffering are less studied (reviewed in Rusch and Gavrilets, 2020). Density-dependent negative feedback (independent of resource availability or the presence of predators; e.g. Boonstra et al., 1998; Edeline et al., 2010; Creel et al., 2013; Mugabo et al., 2017) or positive feedback (Dantzer et al., 2013) through effects on features such as fecundity and body size have been widely studied with population models including social behaviour (e.g. Grimm et al., 2003; Zeigler and Walters, 2014), but the effects of other types of social stressors at the population level have been less considered.

## Conclusions

Here, we have presented an overview of social stress research, giving both a historical perspective and consideration of what we know about social stress throughout the life. This Review has highlighted how the complex social environment can both induce and buffer the stress responses of individuals across multiple time scales: from early life, at adulthood and across generations. There is a rich literature base of examples of such effects of social stress on the stress response itself, as well as on other aspects of phenotype and behaviour (such as social behaviour, learning and anti-predator responses); these effects are underlain by diverse physiological mechanisms and have potential adaptive explanations.

When considering the field of social stress research, it is apparent that a number of outstanding issues remain. In particular, in the future it will be important to more fully integrate studies from laboratory models with field data. We hope that the framework presented above will inform future studies that aim to address these issues. In addition, the field will benefit from new technological advances to measure stress, as well as from developing new theoretical models. Stress responses can allow animals to adapt to rapidly changing environments, and the social environment is a key mediator of these responses in animals across varying degrees of social complexity. Thus, we see this area as ripe for future study and believe that it will benefit from strong integration of experimental biology with eco-evolutionary field and modelling studies.
